# The Effects and Toxicity of Different Pyrene Concentrations on *Escherichia coli* Using Transcriptomic Analysis

**DOI:** 10.3390/microorganisms12020326

**Published:** 2024-02-04

**Authors:** Han Zhu, Linfeng Gong, Ruicheng Wang, Zongze Shao

**Affiliations:** 1 Key Laboratory of Marine Genetic Resources, Third Institute of Oceanography, Ministry of Natural Resources, Xiamen 361005, Chinanmseisvsiecrsan@gmail.com (R.W.); 2 State Key Laboratory Breeding Base of Marine Genetic Resources, Xiamen 361005, China

**Keywords:** *Escherichia coli*, pyrene, transcription, response, transport

## Abstract

Pyrene is a pollutant in the environment and affects the health of living organisms. It is important to understand microbial-mediated pyrene resistance and the related molecular mechanisms due to its toxicity and biodegradability. Due to the unclear response mechanisms of bacteria to PAHs, this study detected the transcriptional changes in *Escherichia coli* under different pyrene concentrations using transcriptome sequencing technology. Global transcriptome analysis showed that the number of differentially expressed genes (DEGs) in multiple metabolic pathways increased with increasing concentrations of pyrene. In addition, the effects and toxicity of pyrene on *Escherichia coli* mainly included the up-regulation and inhibition of genes related to carbohydrate metabolism, membrane transport, sulfate reduction, various oxidoreductases, and multidrug efflux pumps. Moreover, we also constructed an association network between significantly differentially expressed sRNAs and key genes and determined the regulatory relationship and key genes of *Escherichia coli* under pyrene stress. Our study utilized pyrene as an exogenous stress substance to investigate the possible pathways of the bacterial stress response. In addition, this study provides a reference for other related research and serves as a foundation for future research.

## 1. Introduction

Polycyclic aromatic hydrocarbons (PAHs) are a series of aromatic compounds containing two or more benzene rings, which are carcinogenic, teratogenic, and mutagenic [1,2,3]. PAHs are easily enriched in various environments and exhibit a significantly toxic influence on living organisms and ecological systems [3,4,5].

As a type of exogenous stress substance, increases in PAH contents in the environment result in various survival pressures for many life forms, especially microorganisms [6,7,8,9]. In addition, microorganisms require a wide range of stress resistance mechanisms as they need to quickly adapt to environmental factors [8,9,10]. Significant changes in several representative resistance genes in *E. coli* respond to semi-lethal concentrations of PAHs, highlighting the critical role of these genes in responses to stress [11]. Laccase CueO from *E. coli* also exhibits an oxidative capacity for PAH compounds in vitro [12,13]. Several reports have shown that selective stress enrichment of microbial communities with higher concentrations of PAHs affects community structure and distribution, and most strains show tolerance to PAHs [9,14,15]. However, for such microorganisms, studies of their intrinsic regulatory metabolic mechanisms in resisting PAHs should be explored, but there are still few.

Pyrene, a typical compound of PAH pollutant lineages, is a polycyclic aromatic hydrocarbon (PAH) with four benzene rings, and it is commonly used as an indicator for detecting PAHs contamination and in studies of the microbial degradation of PAHs [1,16,17,18,19]. Due to the relatively high molecular weight and number of benzene rings, the degradation of pyrene in the environment is more difficult to analyze than lighter PAH compounds, and its physiological and ecotoxicological toxicity is more obvious [16,19], thus necessitating further research into pyrene.

Previous research has shown that there are various widespread and non-specific anti-stress mechanisms in many bacteria that protect them from external stimuli [8,9,10]. Therefore, aiming to explore the stress response of bacteria to PAHs, we conducted transcriptome analysis of the response to pyrene stress utilizing a key model strain, *Escherichia coli*. After transcriptome sequencing, we characterized the global expression of the response to stress. Moreover, the changes in the growth status of *E. coli* over time under different pyrene concentrations were also identified. Our research presented phenotypic evidence of bacterial response to pyrene stress, revealing the metabolic pathways related to PAH resistance. In addition, this research will provide a preliminary basis for further study on the key regulatory genes and response pathways of stress mechanisms and provide a reference for related research on microorganisms under stress from other exogenous substances.

## 2. Materials and Methods

### 2.1. Bacterial Strain and Culture Conditions

The strain *Escherichia coli* DH5α was previously preserved in our laboratory. This strain is a specially treated mutant strain that mainly manifests as an immune deficiency to exogenous DNA. This strain cannot use PAHs as the sole carbon source for growth. This strain was specifically selected for this experiment to facilitate subsequent molecular experiments.

Pyrene was dissolved in acetone, and the acetone was evaporated after added them to the medium and then mixed well. The gradient culture experiments were conducted using different concentrations of pyrene, ranging from 0 to 1000 mg/L and 1.5 to 10 g/L, in Lysogeny broth (Luria Bertani, containing peptone, 10 g/L; yeast extract, 5 g/L; NaCl, 10 g/L; pH, 7.0) for *E. coli*. The entire culture system was analyzed in an Automatic Microbial Growth Analyzer (FP-1100-C, Bioscreen C, Helsinki, Finland) to measure growth curves. All elements were added to a dedicated 100-well plate before starting; then, the 100-well plate was placed in the Automatic Microbial Growth Analyzer instrument to shake the culture. The OD_600_ value was measured through a transparent part of the plate. Since the content of pyrene in the culture system was fixed, the errors caused by the insolubility of pyrene would be offset. The OD_600_ values were detected using per-hour intervals and oscillate cultures at 37 °C, which is the optimal growth temperature for *E. coli*. Three replicates were conducted, and the average OD_600_ values were calculated for each concentration. After accounting for the OD_600_ values for the substrate effect, we excluded data with larger errors and calculated the average for the OD_600_ values of the parallel group. The processed data were compiled into the growth curves.

### 2.2. RNA Extraction, cDNA Synthesis, and Sequencing

Based on the growth curves, we selected 300, 600, 1000, and 0 mg/L concentration groups for transcriptome sequencing and then analyzed the data. Three replicates were conducted for each concentration, and one copy was kept. After the bacterial growth reached the logarithmic phase, we collected the bacteria using a centrifuge. Total RNA was extracted using TRIzol^®^ reagent according to the manufacturer’s instructions, and DNase I (TaKara, Beijing, China) was used to remove genomic DNA. Then, the RNA quality was measured using a 2100 Bioanalyzer (Agilent Technologies, Waldbronn, Germany) and then quantified using an ND-2000 (NanoDrop Technologies, Thermo Fisher Scientific, East Grinstead, UK). RNA was isolated and purified, whereby rRNA was removed (Ribo Zero Magnetic kit, Epicenter, Madison, WI, USA) after extracting the total RNA. The obtained mRNAs were broken into short fragments (200 nt) by first adding a fragmentation buffer. Then, double-stranded cDNA was synthesized using a SuperScript double-stranded cDNA synthesis kit (Invitrogen, Carlsbad, CA, USA) with random hexamer primers (Illumina, San Diego, CA, USA). A library was obtained after PCR amplification, which can be used for high-throughput sequencing. The Illumina HiSeq sequencing platform was used to sequence the transcriptome, and the generated data were analyzed via bioinformatics. All analyses were conducted using the free online program Majorbio Cloud Platform (www.majorbio.com, accessed on 13 September 2022) from Shanghai Major Bio-pharm Technology Co., Ltd. (Shanghai, China) RNA-Seq reads were deposited in GenBank under accession numbers SAMN38428292 to SAMN38428303.

### 2.3. Data Quality Control and Enrichment Analyses

The high-quality reads and clean data were obtained by removing reads with adaptors (Supplementary Table S1). High-quality reads were mapped to the reference genome. Serial genes can be measured via RSEM software (version 1.2.31) by counting the expression levels of genes and transcripts using single or double-ended sequencing data [20]. The expression values were measured using FPKM methods and can be directly used to compare the differences between samples [21]. Differentially expressed genes were analyzed using the DESeq2 with log_2_FoldChange ≥ 1 and *p*-value ≤ 0.05 as the difference threshold, and the genes with significant differences were obtained through analysis. The correlation was further checked between different samples, and the duplicates with poor correlation were eliminated. The function of each differentially expressed gene (DEG) was analyzed using the GO database (Gene Ontology, http://www.geneontology.org/, accessed on 27 February 2023) and the Kyoto Encyclopedia of Genes and Genomes database (KEGG, http://www.genome.jp/kegg/, accessed on 27 February 2023). The *p*-value was calibrated using four multiple testing methods (Bonferroni, Holm, Sidak, and false discovery rate) to control the false positive rate. Typically, when the corrected *p*-value (p-fdr) ≤ 0.05, the GO and KEGG enrichment pathways were defined as significantly enriched pathways.

### 2.4. Quantitative Real-Time PCR Analyses and Statistical Analyses

Five genes were chosen to verify RNA-seq data via qRT-PCR after obtaining the counts of DEGs in *E. coli* (Supplementary Table S2). Total RNAs and cDNAs were extracted via the abovementioned methods, and qRT-PCR was conducted according to a previously reported method [22]. The significant differences among groups were subjected to one-way analysis of variance (ANOVA) [23] and multiple comparisons using SPSS 19.0 software (SPSS Inc., Chicago, Illinois, USA). Statistical significance was defined as *p* < 0.05 (indicated by * in Supplementary Figure S1) or *p* < 0.01 (indicated by ** in Supplementary Figure S1).

## 3. Results

### 3.1. Growth Curves of E. coli under Different Pyrene Concentrations

**Figure 1:**
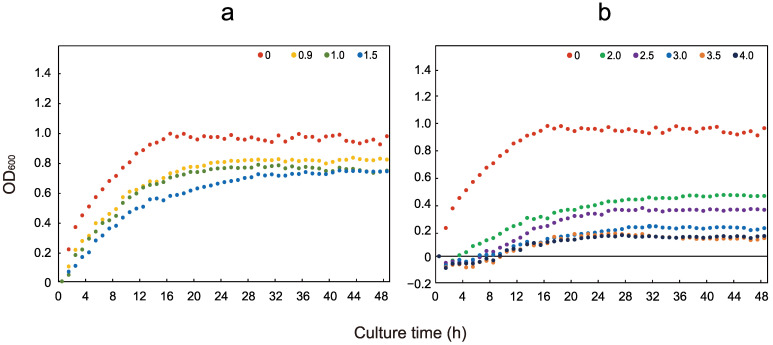
Growth curves for *E. coli* under different pyrene concentrations. (**a**): The growth curves of *E. coli* under 0.9–1.5 g/L; (**b**): The growth curves of *E. coli* under 2.0–4.0 g/L. The pyrene concentrations in the experiment were divided into four concentration ranges: 1 to 800 mg/L, 0.9 to 1.5 g/L, 2.0 to 4.0 g/L, and 5.0 to 10.0 g/L. The ranges of 1 to 800 mg/L and 5.0 to 10.0 g/L are shown in Supplementary Figure S2.

We measured the OD_600_ values of *E. coli* growth under different pyrene concentrations and plotted them as a set of curves. Through analyzing the growth curves, we observed that *E. coli* exhibited growth at concentrations ranging from 0 to 4.0 g/L. At concentrations from 1 to 800 mg/L (Supplementary Figure S2), the growth was similar to that of the control group (0 mg/L). There was a slight change when the concentration of pyrene exceeded 800 mg/L, and the growth condition decreased with increasing concentrations within the range of 900 mg/L to 4.0 g/L (Figure 1). At concentrations exceeding 5 g/L, growth was essentially completely halted (Supplementary Figure S2).

### 3.2. Summary of the Differentially Transcribed Genes

We constructed twelve cDNA libraries, including the control group of *E. coli* and the experimental groups with three different concentrations. The Q30 base percentage of each sample is higher than 93.53% (Supplementary Table S1). We obtained the gene expressions of *E*. *coli* via high-throughput RNA sequencing, with a total of 4714 genes expressed and 1368 significant DEGs found in all concentrations. By comparing the above genes with the GO and KEGG databases, we found that the GO annotation rate was 82.31%, and the KEGG annotation rate was 71.28%.

We analyzed the differences in gene expression among the experimental groups with varying concentrations of pyrene. The results showed that 620, 846, and 1127 DEGs were significantly expressed at 300, 600, and 1000 mg/L, respectively (Supplementary Figure S3a). At 600 mg/L, there were more significantly up-regulated and down-regulated genes than the other two concentrations. Moreover, the levels of significant up-regulation and down-regulation at 1000 mg/L were higher than those at 300 mg/L (Supplementary Figure S3b), indicating that the physiological response of *E. coli* was stronger at a concentration of 1000 mg/L than at 300 mg/L. The distribution of the volcano plot fold change indicates that the most significantly changed genes among the DEGs are distributed as 1 ≤ |Log2FC| ≤ 4.

### 3.3. qRT-PCR Validation of DEGs

With the aim of further verifying the reliability of the transcriptome results, we selected five differentially expressed genes for qRT-PCR (Supplementary Table S2). Among the groups, the NJ74_RS04375, NJ74_RS09325, NJ74_RS21725, NJ74_RS12255, and NJ74_RS18825 genes were generally up-regulated. Most up-regulation levels for genes in *E. coli* were in the range of 1.0 to 2.0 fold, and the highest up-regulation levels were up to 5.0. Furthermore, qRT-PCR quantification of *E. coli* was similar to the gene expression trends and indicates that the expression patterns of transcriptomes are reliable (Supplementary Figure S1).

### 3.4. Annotation Analysis of Global Metabolic Pathways for E. coli

**Figure 2:**
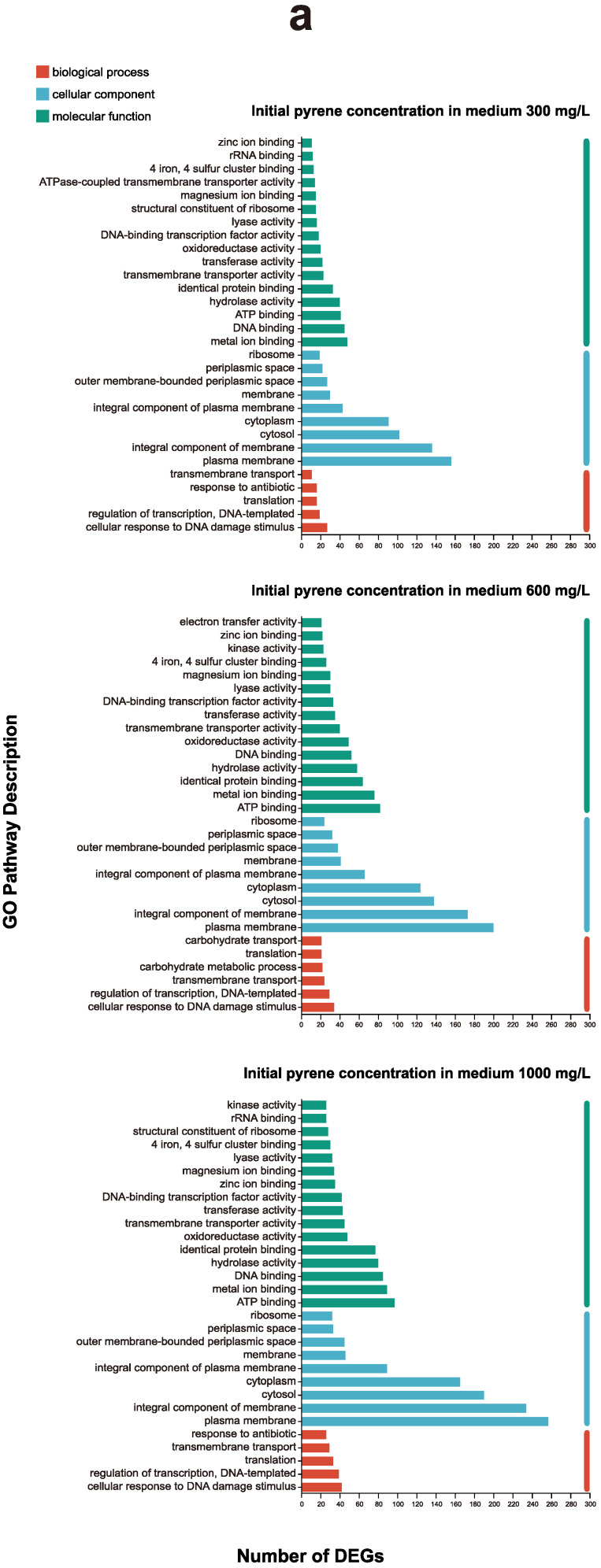

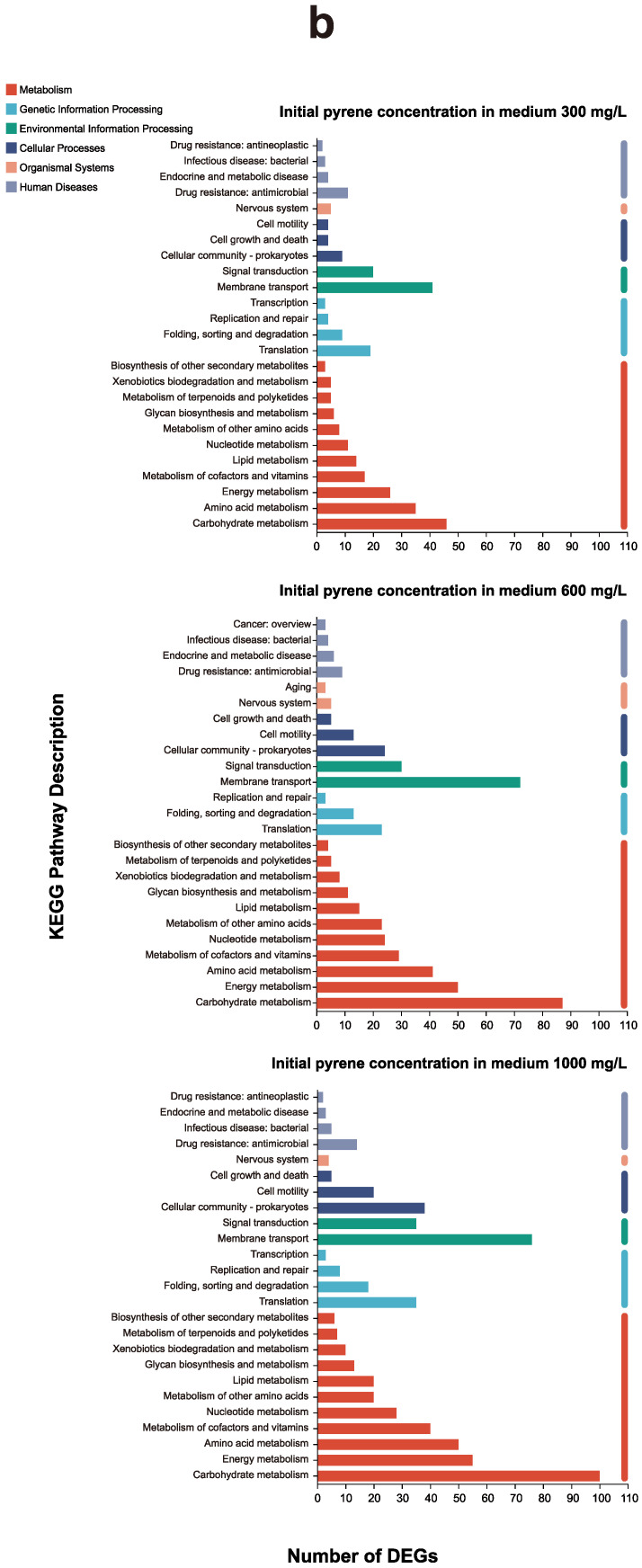
(**a**) GO annotation of significant DEGs in *E. coli.* (**b**) KEGG annotation of significant DEGs in *E. coli*.

Aiming to characterize the global metabolic differences, we compared various metabolic pathways with the highest number of regulated genes in *E. coli*, as well as the top 30 terms in GO enrichment and 25 terms in KEGG enrichment (Figure 2). The GO annotation results showed that the vast majority of pathways were reflected under all three concentrations for *E. coli*, and the pathways were also consistent at different concentrations (Figure 2a). The key metabolic pathways of the KEGG terms were also consistent in the three experimental groups (Figure 2b).

### 3.5. Annotation Analysis of Differentially Regulated Gene Expression for E. coli under All Concentrations

**Figure 3:**
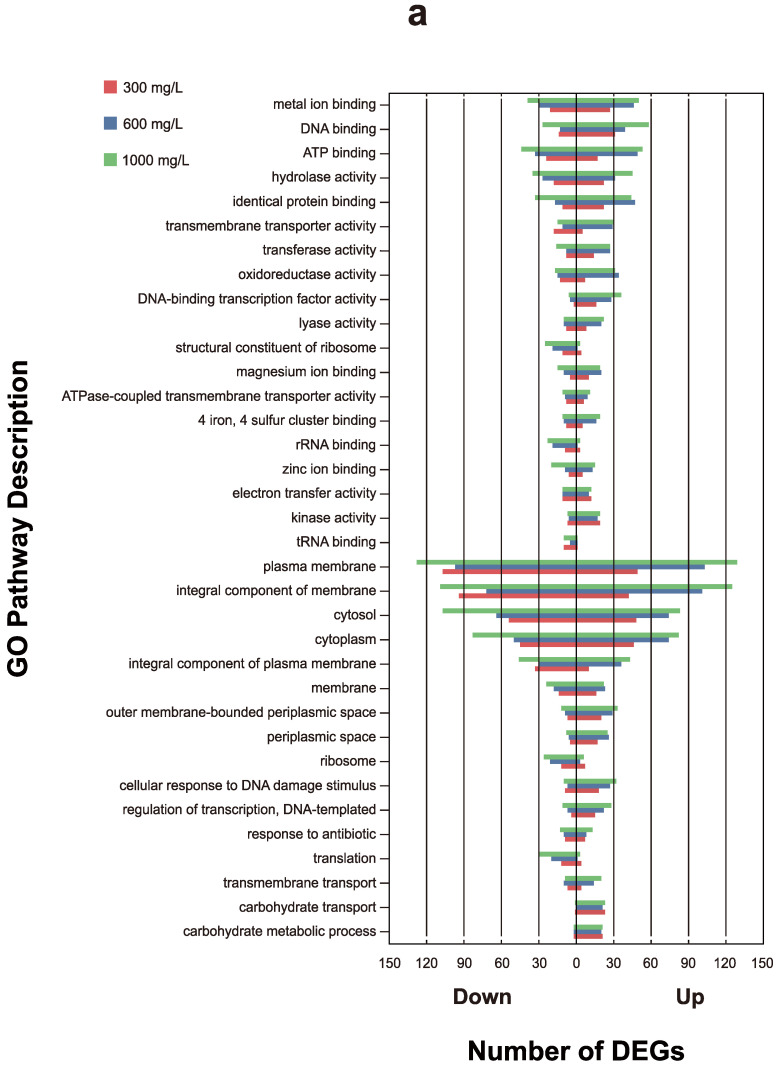

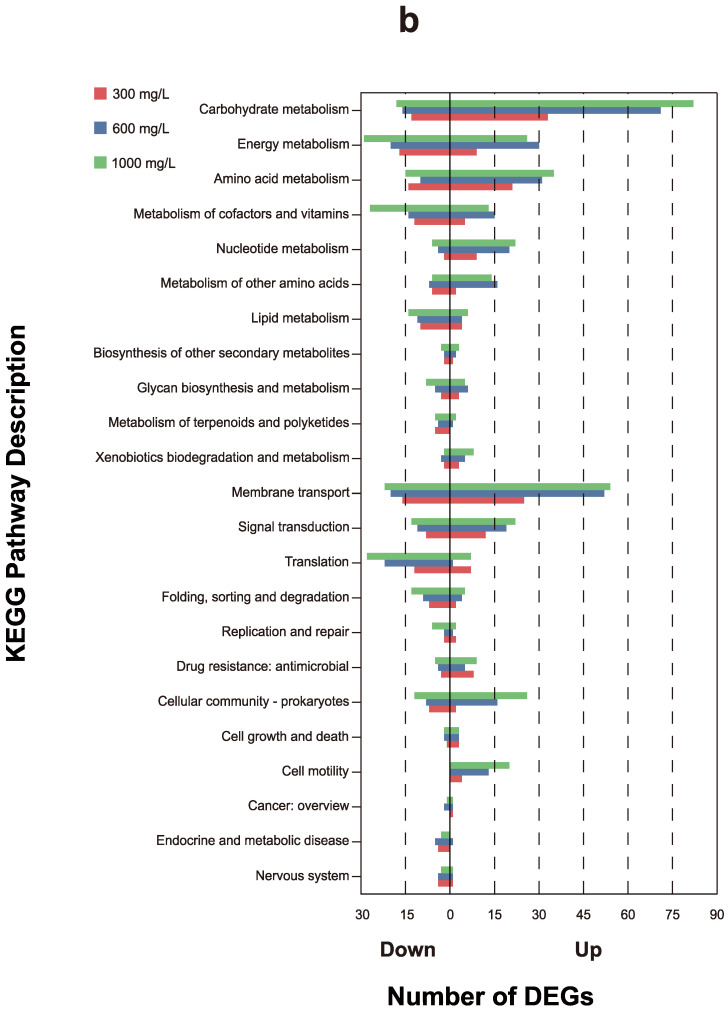
(**a**) Number of DEGs for GO functional classification of *E. coli*. The metabolic pathway with a positive value on the horizontal axis represents the up-regulated genes, while negative value represents the down-regulated genes. (**b**) Number of DEGs for KEGG functional classification of *E. coli*.

Aiming to compare the similarities and differences in metabolic pathways exposed to different concentrations of pyrene, we distinguished the annotation results of significantly expressed genes in *E. coli* (Figure 3). Most metabolic pathways for *E. coli* had more up-regulated genes than down-regulated genes, and several pathways had almost no down-regulated expression genes. Various metabolic pathways were inhibited by high concentrations of pyrene. The various experimental groups exhibited similar counts of up-regulated genes, while there was a positive correlation between the number of down-regulated genes and concentration. The most pronounced inhibition occurred at a concentration of 1000 mg/L. Various pathways responded to pyrene, and the number of up-regulated genes also increased in these pathways as the concentration increased.

### 3.6. Network Analysis of sRNA in E. coli under Different Pyrene Concentrations

A total of 180 sRNAs were identified in *E*. *coli*, ranging in length from 30 to 187 nucleotides (Supplementary Table S3). In the sRNA expressed in this study, there were 11, 14, and 18 significantly expressed genes at 300, 600, and 1000 mg/L, respectively. In detail, there were 2, 0, and 7 up-regulated expressions, as well as 9, 14, and 11 down-regulated expressions at 300, 600, and 1000 mg/L, respectively (Supplementary Table S4). To further evaluate the function of significantly different expressions of sRNA target genes, we identified the part of the target gene linked to sRNA with the highest degree of up-regulation (|Log_2_FC| ≥ 3) and then performed GO and KEGG enrichment on these target genes (Supplementary Table S5). We screened for the above sRNA target genes at all concentrations and analyzed them through the GO and KEGG enrichment. The results show that among the abovementioned target genes, the main terms include carbohydrate metabolism, energy metabolism, and membrane transport. In addition, these genes are also involved in nucleotide metabolism, the metabolism of other amino acids, the biosynthesis of other secondary metabolites, the metabolism of cofactors and vitamins, glycan biosynthesis and metabolism, lipid metabolism, and amino acid metabolism pathways.

**Figure 4:**
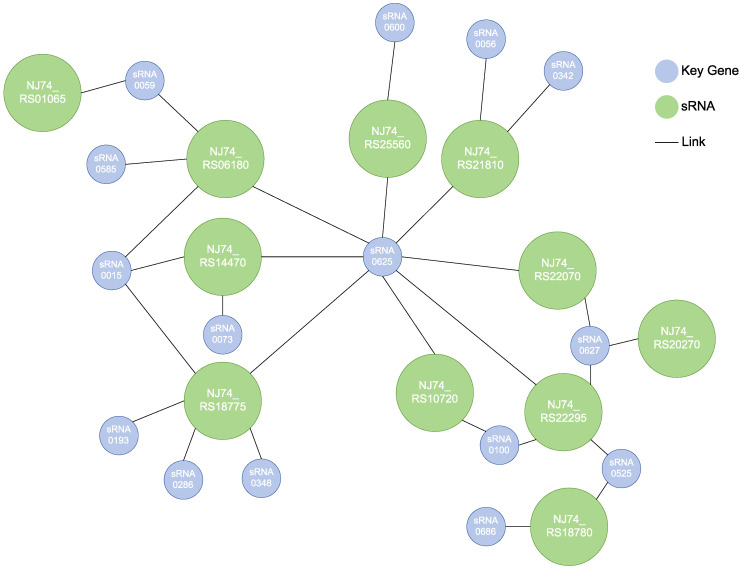
The interaction network of key genes and sRNAs in *E. coli*.

Based on the corresponding relationship between different sRNAs and target genes, we constructed a regulatory network of stress responses in *E. coli* under different pyrene concentrations (Figure 4). This network mainly focuses on 11 key mRNA genes and analyzes their associations and interactions with differentially expressed sRNAs. Based on this network diagram, we can identify three key genes with the highest degree of network connectivity (NJ74_RS06180, NJ74_RS18775, and NJ74_RS22295) and one central position sRNA (sRNA0625). Further analysis revealed that these three genes are adenylyl sulfate kinase, sulfate/thiosulfate ABC transporter permease, and RHS domain-containing protein. These three key genes are all linked to sRNA0625.

### 3.7. Carbohydrate Metabolism, Energy Metabolism, and Oxidoreductase in E. coli

After GO and KEGG enrichment of the sRNA target genes, we found that various target genes were enriched in carbohydrate metabolism. Among all DEGs, the number of DEGs in carbohydrate metabolism is also one of the highest. Considering that carbohydrate metabolism may provide a material basis for resistance to pyrene stress in *E. coli*, we further analyzed the DEGs of this pathway. The DEGs of carbohydrate metabolism are mainly enriched in butanoate metabolism, pyruvate metabolism, glyoxylate and dicarboxylate metabolism, and glycolysis/gluconeogenesis pathways (Supplementary Table S6). Most genes in carbohydrate metabolism are up-regulated, and the level of up-regulation is concentrated from 1.0 to 4.0 fold (Supplementary Table S7). There is a total of 25 genes up-regulated more than 5.0 fold (NJ74_RS00655, NJ74_RS17475, NJ74_RS04535, novel0293, NJ74_RS01070, novel0385, NJ74_RS11870, NJ74_RS08950, NJ74_RS04260, NJ74_RS11935, novel0132, NJ74_RS11920, NJ74_RS09985, NJ74_RS12985, NJ74_RS12400, NJ74_RS10665, novel0325, NJ74_RS07890, NJ74_RS12055, NJ74_RS21540, NJ74_RS17855, NJ74_RS04415, novel0200, NJ74_RS12045, NJ74_RS07895; Table 1).

**Figure 5:**
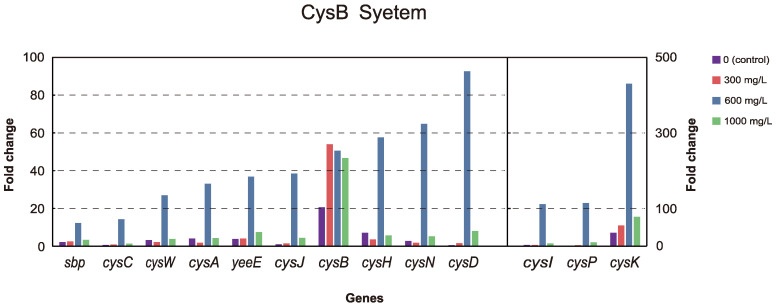
The DEGs of the CysB system in *E. coli*. The vertical axis represents the number of gene copies within a range of 0 to 100 on the left axis and 0 to 500 on the right axis.

With regard to energy metabolism, many genes that were mostly up-regulated in this pathway belong to the sulfate reduction pathway and are regulated via the *CysB* gene. The *CysB* system, a complete metabolic pathway from extracellular sulfate transport to cysteine synthesis, exhibited up-regulation of key genes in this process in *E. coli*. The genes of this pathway were up-regulated at all concentrations, but the counts and levels of up-regulation were particularly significant at 600 mg/L (Figure 5). The collective expression of sulfate reduction pathway up-regulation may point to the oxidation of PAHs. We further analyzed the DEGs that belong to oxidoreductase and related genes in *E. coli*. The results showed that although there were not very high levels of up-regulation of oxidoreductase genes, the number of up-regulated genes was significantly higher than that of down-regulated genes, and the upward trend was very obvious (Supplementary Table S8).

### 3.8. Analysis of Membrane Transport, SoxRS System, and Efflux Pumps in E. coli

According to the enrichment results, the number of DEGs in the membrane transport pathway is second only to carbohydrate metabolism. Among the genes in membrane transport, ABC transporters account for the largest proportion, and the genes in this pathway are mainly responsible for transporting nutrients (Supplementary Table S9). In this experiment, the up-regulated genes in this pathway mainly function to transport multiple carbon sources into the cell. Another result of membrane transport is that a portion of DEGs belong to the bacterial secretion system pathway, and the up-regulated genes in this pathway are type II secretion system protein and translocase (Supplementary Table S10). The genes in the phosphotransferase system (PTS) also showed significantly differential expression and were all up-regulated. Further analysis of the up-regulation revealed that the genes in this pathway were mainly up-regulated from 2.0 to 4.0 fold, with only three genes (NJ74_RS04255, NJ74_RS03620, and NJ74_RS09985) up-regulated from 5.0 to 6.0 fold (Supplementary Table S11).

In addition to the aforementioned pathways, the SoxRS system has also been shown to be associated with oxidative stress processes in many studies [24]. In this experiment, there were also various DEGs in the SoxRS system of *E. coli* (Supplementary Table S12). More than half of the DEGs of the SoxRS system in *E. coli* were transport genes, which were mainly drug-resistance-related in addition to nutrient transporters, including RND efflux pumps, Tol system genes, MFS transporters, multidrug efflux genes, and TonB genes (Supplementary Table S13).

## 4. Discussion

According to GO and KEGG enrichment (Figure 2), DEGs increased with concentration in the metabolic terms dominated by carbohydrate metabolism, which is consistent with the expected pattern. Figure 3 shows that the number of up-regulated and down-regulated genes both increase with increasing concentration. A possible explanation is that, as the concentration increases, a greater number of genes are inhibited, and the stress response of *E. coli* to PAHs is concurrently increased.

In detail, the stress response of *E. coli* to pyrene involved multiple metabolic pathways. Primarily, carbohydrate metabolism and other fundamental metabolic pathways are involved. The number of these up-regulated genes increased with increasing pyrene concentrations, indicating that, as the response increases, material synthesis and energy consumption also synchronously increase. The number of up-regulated and down-regulated genes reached their maximum value when the concentration reached 1000 mg/L. There was a similar number of up-regulated genes in certain pathways among the three concentrations, such as pyruvate metabolism, glycolysis and gluconeogenesis, and glyoxylate and dicarboxylate metabolism, suggesting that these pathways play an important role in *E. coli*. Compared to 300 mg/L of pyrene, the number of up-regulated genes at 1000 mg/L was closer to 600 mg/L, and the carbohydrate metabolism response for the 600 and 1000 mg/L groups was similar. This suggests that when PAHs increase from low to high concentrations, the pressure caused by PAHs on *E. coli* is not stable. It is possible that stress, similar to the growth curve of bacteria, may first slow down, followed by a rapid increase, reaching the upper limit of *E. coli*’s ability after exceeding a certain concentration, with no further changes or minimal changes afterward.

The sulfate reduction pathway plays a crucial role in the oxidation process of many substances [25]. Previous reports have indicated that the entry of PAHs into cells can trigger oxidative stress [26]. The CysB system is mainly responsible for sulfate reduction and the synthesis of cysteine. It indirectly affects cellular oxidative stress through the reducibility of cysteine and its role as the active site in key proteins [27]. The redox process via oxidoreductases is one of the key steps in the degradation of PAHs [28,29]. Most redox-functional enzymes in *E. coli* contain proteins with Fe-S clusters, and the active sites of most dioxygenase and oxide-reductase in this experiment are Fe-S clusters [30]. Relevant reports have suggested that when *E. coli* is exposed to PAHs, *recA*, *katG*, *fabA*, and *grpE* genes, there is significant up-regulation for representatives of DNA, oxidation, membrane, and protein damage [11].

Previous reports have shown that the transport system of bacteria is closely related to stress [31]. Our research indicates that three pathways of membrane transport and multidrug efflux pumps related to transporter activity are significantly enriched and up-regulated at all concentrations, using GO functional enrichment and KEGG enrichment. According to various reports, the transporter-related genes of *Lactobacillus plantarum* were significantly differentially expressed under acid stress, which is consistent with the results of this study [32]. In this study, DEGs associated with transport genes were mainly divided into membrane transport and multidrug efflux pumps. The membrane transport-related pathways also include ABC transporters, bacterial secretion systems, and PTS transport proteins. The genes related to ABC and PTS transporters in *Lactobacillus plantarum* exhibit significant changes under acid stress conditions [32]. For the genes of the SoxRS system responding to pyrene, efflux genes represented by RND transporters were observed. Parts of RND efflux pumps and other multidrug efflux genes outside the Sox system were both significantly up-regulated in *E. coli*. RND efflux pumps are bacterial drug-resistance mechanisms that efflux a variety of hazardous substances to the outside of the cell, and there are five types of efflux pumps in *E. coli* [33,34,35,36,37]. It has previously been reported in the literature that the RND exocytosis pump plays a key role in the organic solvent tolerance and multidrug resistance of bacteria [38,39]. A previous study on PAH stress in *Pseudomonas aeruginosa* has shown that bacterial efflux pumps and transport genes play a key role in bacterial resistance to organic solvent toxicity [40]. The up-regulation of genes in other drug-resistant exocytosis proteins is also required in *E. coli* for an auxiliary response due to the low number of RND exocytosis pumps. The centralized up-regulation of the genes of this family also demonstrates their roles in cells exposed to pyrene stress. The main function of bacterial secretion systems and multidrug efflux pumps may be to transport intracellular PAHs from bacteria to the outside of the cell to prevent damage.

sRNAs are a regulatory factor involved in gene expression in organisms. Under adverse conditions, organisms will respond to stress by regulating gene transcription levels or producing new proteins through sRNA [41]. We found that differentially expressed sRNA target genes contain multiple genes related to carbohydrate metabolism and membrane transport. Previous reports have also mentioned that these target genes regulated via sRNA contain multiple genes associated with carbohydrate metabolism, membrane transport, and other metabolic pathways [42]. According to the association network between key genes and sRNA, many sRNAs are key regulatory factors of the stress response in *E. coli*. Moreover, various key genes associated with sRNA, such as *cysC* (NJ74_RS06180), *cysT* (NJ74_RS18775), and *tsuB* (NJ74_RS14470), also play important roles in the stress response.

## 5. Conclusions

The adaptation of bacteria to external environmental pressure is often accompanied by transcriptional regulation. This study investigates the transcriptional changes in *E. coli* under different concentrations of pyrene, with a focus on elucidating the key metabolic pathways involved in its response to pyrene stress. This study provides evidence for the expression patterns of mRNAs and sRNAs in *E. coli* exposed to different concentrations of pyrene. In our study, significant up-regulation of genes related to transport was observed, indicating that *E. coli* may avoid damage by transporting pyrene out of the cell. In addition, we also investigated key genes associated with significantly differentially expressed sRNAs, which could serve as potential evidence for the involvement of the sulfate reduction pathway in the oxidation of PAHs. Moreover, various oxidoreductases in *E. coli* may also oxidize PAHs, which could be a mechanism by which *E. coli* responds to pyrene stress. Transcriptomic analysis can be used to determine changes in mRNAs and sRNAs induced by different concentrations of pyrene, laying the foundation for future research. However, this study lacks further validation experiments of these key genes; therefore, further study will be carried out in the future.

## Figures and Tables

**Table 1:** The genes with the highest degree of up-regulation in *E. coli*.

Gene_Id	Gene Name	Gene Description
NJ74_RS00655	*rpiB*	Ribose 5-phosphate isomerase B
NJ74_RS17475	*ilvM*	Acetolactate synthase II small subunit
NJ74_RS04535	*ilvB*	Acetolactate synthase I/II/III large subunit
novel0293	-	Tartrate dehydrogenase/decarboxylase/D-malate dehydrogenase
NJ74_RS01070	*aceB*	Malate synthase
novel0385	-	Deoxyribose-phosphate aldolase
NJ74_RS08950	*lacZ*	Beta-galactosidase
NJ74_RS04260	*bglB*	6-phospho-beta-glucosidase
NJ74_RS11870	*glnA*	Glutamine synthetase
NJ74_RS11935	*yihU*	4-hydroxybutyrate dehydrogenase/sulfolactaldehyde 3-reductase
novel0132	-	Aconitate hydratase 2/2-methylisocitrate dehydratase
NJ74_RS11920	*yihR*	Aldose 1-epimerase
NJ74_RS09985	*agaV*	N-acetylgalactosamine PTS system EIIB component
NJ74_RS12985	*ydiF*	Acetate CoA-transferase
NJ74_RS12400	*rspA*	Mannonate dehydratase
NJ74_RS10665	*aldA*	Lactaldehyde dehydrogenase/glycolaldehyde dehydrogenase
NJ74_RS09990	*kbaZ*	D-tagatose-1,6-bisphosphate aldolase subunit GatZ/KbaZ
novel0325	-	Formate dehydrogenase major subunit
NJ74_RS07890	*gatZ*	D-tagatose-1,6-bisphosphate aldolase subunit GatZ/KbaZ
NJ74_RS12055	*rhaB*	Rhamnulokinase
NJ74_RS17855	*fadB*	3-hydroxyacyl-CoA dehydrogenase/enoyl-CoA hydratase/3-hydroxybutyryl-CoA epimerase/enoyl-CoA isomerase
NJ74_RS04415	*dgoD*	Galactonate dehydratase
NJ74_RS12045	*rhaD*	Rhamnulose-1-phosphate aldolase
NJ74_RS07895	*gatY*	Tagatose 1,6-diphosphate aldolase GatY/KbaY
novel0200	-	Fumarate reductase flavoprotein subunit

## Data Availability

Data are contained within the article and Supplementary Materials.

## Supplementary Materials

The following supporting information can be downloaded at https://www.mdpi.com/article/10.3390/microorganisms12020326/s1: Figure S1: The qRT-PCR results for five genes in *E. coli*; Figure S2: Growth curves for *E. coli* at 1–800 mg/L and 5.0–10.0 g/L pyrene concentrations; Figure S3: Volcano and Venn plots of DEGs in *E. coli* at different concentrations; Table S1: The data sequencing quality for *E. coli*; Table S2: The primers for qRT-PCR; Table S3: The data for sRNAs in *E. coli*; Table S4: The significantly different expression of sRNAs in *E. coli*; Table S5: The DEGs linked to differentially expressed sRNAs in *E. coli*; Table S6: The number of up-regulated and down-regulated genes in different carbohydrate metabolism pathways in *E. coli*; Table S7: The DEGs of carbohydrate metabolism in *E. coli*; Table S8: The DEGs of oxygenase and Fe-S cluster protein in *E. coli*; Table S9: The DEGs of ABC transporters in *E. coli*; Table S10: The DEGs of the bacterial secretion system in *E. coli*; Table S11: The DEGs of the phosphotransferase system in *E. coli*; Table S12: The DEGs of the SoxRS system in *E. coli*; Table S13: The DEGs of efflux pumps in *E. coli*.
